# Association between Dietary Protein Intake, Regular Exercise, and Low Back Pain among Middle-Aged and Older Korean Adults without Osteoarthritis of the Lumbar Spine

**DOI:** 10.3390/jcm11051220

**Published:** 2022-02-24

**Authors:** Hye-Mi Noh, Yi Hwa Choi, Soo Kyung Lee, Hong Ji Song, Yong Soon Park, Namhyun Kim, Jeonghoon Cho

**Affiliations:** 1 Department of Family Medicine, Hallym University Sacred Heart Hospital, College of Medicine, Hallym University, Anyang 14068, Korea; hyeminoh@hallym.or.kr (H.-M.N.); hongji@hallym.or.kr (H.J.S.); 2 Department of Anesthesiology and Pain Medicine, Hallym University Sacred Heart Hospital, College of Medicine, Hallym University, Anyang 14068, Korea; agneta@hallym.or.kr (S.K.L.); alisia94@hallym.or.kr (N.K.); chojh137@hallym.or.kr (J.C.); 3 Department of Family Medicine, Chuncheon Sacred Heart Hospital, College of Medicine, Hallym University, Chuncheon 24253, Korea; pyongs@hallym.or.kr

**Keywords:** exercise, low back pain, older adults, protein intake, physical activity, KNHANES

## Abstract

This study aimed to evaluate the effect of dietary protein intake and regular exercise on low back pain (LBP) using data from the Korea National Health and Nutrition Examination Survey. A total of 2367 middle-aged and older adults (≥50 years) who underwent dual-energy X-ray absorptiometry and plain radiography of the lumbar spine were included. LBP was defined using a questionnaire to determine the presence of LBP lasting more than 30 days in the preceding three months. Twenty-four-hour dietary recall data were used to estimate protein intake, and regular exercise was assessed using the International Physical Activity Questionnaire. Multivariable logistic regression analysis revealed that men who did not perform regular exercise had a high probability of LBP (odds ratio [OR] 2.34; 95% confidence interval [CI] 1.24–4.44). Low protein intake (<0.8 g/kg/day) was associated with high odds for LBP in women (OR 1.83; 95% CI 1.12–2.99). Low protein intake and lack of regular exercise were also associated with a higher probability of LBP in women (OR 2.91; 95% CI 1.48–5.72). We recommend that women over 50 years of age consume the recommended daily amount of protein to prevent LBP and engage in regular exercise.

## 1. Introduction

Low back pain (LBP) is a common condition with a reported prevalence of 1.0% to 58.1% [1]. The medical costs attributable to LBP are estimated to be USD 26 billion, and the additional cost of productivity lost because of LBP is estimated to be USD 23.5 billion [2]. Chronic pain in older adults, which is commonly associated with musculoskeletal disorders, results in adverse health outcomes, including disability, falls, depression, insomnia, and social isolation [3].

LBP is also associated with a sedentary lifestyle. A recent systematic review and meta-analysis reported that strengthening by stretching or aerobic exercises helped to prevent LBP in both the general and working populations [4]. Exercise therapy was found to be effective in reducing pain and improving functionality in patients with non-specific LBP in the absence of specific pathology [5,6]. However, older patients encounter difficulty when trying to incorporate exercise into their daily lives in the real world due to frailty and poor performance caused by low muscle strength, comorbidities, and progressive degenerative changes in multiple joints.

Dietary protein intake can sustain muscle mass and physical function and prolong independent living in the older population [7,8]. Intake of sufficient dietary protein enables preservation or an increase in muscle mass resulting from a positive balance between synthesis and breakdown of muscle protein in older adults [9,10]. Many patients with LBP and their caregivers have questions concerning nutritional requirements and supplements in clinical practice. However, considering the level of interest among affected individuals, few studies have focused on daily dietary intake in patients with chronic LBP [11,12]. To date, the association between dietary protein intake and regular exercise in older patients with chronic LBP has been unclear.

Lifestyle factors, such as physical activity and nutrition, may be associated with LBP in the aging population. However, there is limited relevant literature in the Asian context. This study aimed to evaluate the association between dietary protein intake, regular exercise, and LBP in the absence of osteoarthritis of the lumbar spine, using a nationally representative sample of middle-aged and older Korean adults.

## 2. Materials and Methods

### 2.1. Study Population

Data from the Fifth Korea National Health and Nutrition Examination Survey (KNHANES V-1 and V-2) collected in 2010–2011 by the Korea Centers for Disease Control and Prevention (KCDC) were analyzed in this study. The KNHANES is a nationwide cross-sectional survey that has been conducted since 1998 by the KCDC. The KCDC selected 21,527 individuals using a complex, stratified, multistage probability sampling design. A total of 17,476 study participants completed the survey, giving a response rate of 81.2%. The present study included 3988 participants (aged ≥50 years) who underwent dual-energy X-ray absorptiometry (DEXA) and plain radiography of the lumbar spine. We excluded subjects who had a specific pathology causing LBP, namely those with radiographic evidence of osteoarthritis (OA) on a lumbar radiograph (n = 1327) and those with a history of vertebral fracture (n = 15). Participants with no response to questionnaire items on LBP (n = 34) or missing data for dietary protein intake (n = 244) or body weight (n = 1) were also excluded. Finally, data for 2367 subjects were included in the study. The 2010 and 2011 KNHANES were approved by the Institutional Review Board of the Korea Centers for Disease Control and Prevention (2010-02CON-21-C and 2011-02CON-06-C, respectively). Written informed consent was obtained from all study participants.

### 2.2. Data Collection and Measurements

Face-to-face interviews and standardized health examinations were conducted. The questionnaire included age, sex, educational attainment, occupation, household income, disease status, and lifestyle risk factors. Smoking status was classified into three categories: current smoker (more than five packs of cigarettes during lifetime and currently smokes every day), ex-smoker (smoked a month ago but has now quit), and never-smoker. Alcohol consumption was defined as consuming alcohol at least once every month over the past year. Physical activity was evaluated using the International Physical Activity Questionnaire [13]. We defined regular exercise as follows: aerobic exercise/weight training/stretching exercise on at least two days in a week or walking as a physical activity for at least 30 min on five days in a week. Aerobic exercise included swimming, doubles tennis, volleyball, badminton, table tennis, and occupational or sports activities, such as carrying objects, except for walking. Weight training consisted of push-ups, sit-ups, lifting dumbbells or barbells, and pull-up bars. Stretching exercise included back stretching, relaxation exercises, and calisthenics. Past medical history, including chronic diseases and vertebral fractures, was assessed using a self-report questionnaire. Chronic diseases included hypertension, diabetes mellitus, liver cirrhosis, chronic kidney disease, cancer, rheumatoid arthritis, and major depressive disorder.

### 2.3. Low Back Pain

We defined study participants as having LBP if they answered “yes” to the question “Have you suffered LBP for more than 30 days during the past three months?”

### 2.4. Radiographic Examination and Body Composition Measurements

Plain radiographs (anteroposterior and lateral views) of the lumbar spine were obtained using an SD 3000 Synchro Stand (Accele Ray SYFM Co., Seoul, Korea). Two musculoskeletal radiologists independently evaluated the severity of OA in all facet joints using the Kellgren–Lawrence grading system. The radiologic grade was classified using the method devised by Yoshimura et al. [14]: 0, normal (no abnormalities including slight osteophytes); 1, suspicious (clear osteophytes); and 2, abnormal (stenosis, osteosclerosis, and large osteophytes). If there was a difference in the radiologists’ grades, the higher grade was selected. The inter-rater agreement was 92.8% [15]. Body composition and bone mineral density were measured by DEXA (DISCOVERY-W, Hologic Inc., Marlborough, MA, USA). Muscle mass was calculated as the difference between lean body mass and bone mineral content, and appendicular skeletal muscle mass (ASM) was defined as the sum of the values recorded for the upper and lower extremities bilaterally. The ASM index was calculated as ASM/(height [m])^2^. Low skeletal muscle mass was defined according to the 2019 Asian Working Group for Sarcopenia consensus statement; the cut-off value for diagnosis of low muscle mass as sarcopenia is <7.0 kg/m^2^ in men and <5.4 kg/m^2^ in women [16]. According to the World Health Organization criteria, bone mineral density of the lumbar spine is defined as normal (T-score ≥ −1.0), osteopenia (−2.5 < T-score < −1.0), or osteoporosis (T-score ≤ −2.5) [17].

### 2.5. Assessment of Dietary Intake

The frequency of dietary intake and amount of each food item were evaluated using a semi-quantitative food frequency questionnaire, with verified validity for 112 food items. Dietary intake was assessed using a 24 h dietary recall to determine the average intake frequency and average daily intake per serving during the preceding year. Trained dietitians helped participants recall their dietary information, such as consumed food, amount, and recipes. The total energy of the dietary intake and each nutrient was calculated using the National Standard Food Composition Table developed by the Rural Development Administration [18]. The daily intake of energy and nutrients for each individual was calculated based on the sum of all the items consumed. Protein intake was classified according to the recommended daily allowance (RDA) for dietary protein (0.8 g/kg/day) as low (<0.8 g/kg/day) or good (≥0.8 g/kg/day) [19].

### 2.6. Statistical Analysis

Complex sample analysis was performed for all the statistical analyses. Continuous variables are presented as the mean ± standard error and compared between groups using the Student’s *t*-test. Categorical data are shown as the estimated percentage (standard error) and compared using the chi-squared test. We performed multivariable logistic regression analysis to estimate the associations between protein intake, regular exercise, and LBP. We divided the subjects into four groups according to protein intake (low, <0.8 g/kg/day; good, ≥0.8 g/kg/day) and regular exercise status (specified exercises or walking for physical activity) as follows: low protein intake with exercise, good protein intake with exercise, low protein intake without exercise, and good protein intake without exercise. The good protein intake with exercise group was defined as the reference group; crude odds ratios (ORs) for LBP were calculated for the other three groups. Age and sex were adjusted in model 1. Body mass index, factors associated with socioeconomic status (household income, occupation, and education level), and lifestyle factors (smoking status and alcohol consumption) were additionally included in model 2. Bone mineral density of the lumbar spine (normal, osteopenia, and osteoporosis), Kellgren–Lawrence grading of the lumbar spine (normal or grade 1), comorbidities, and total energy intake were added to the final model (model 3). All statistical analyses were performed using SPSS software (version 20.0; IBM Corp., Armonk, NY, USA). A *p*-value < 0.05 was considered statistically significant.

## 3. Results

The study sample consisted of 1047 men and 1320 women. The mean age (standard error) was 59.4 years (0.34) in men and 59.7 years (0.30) in women. Table 1 shows the demographic and clinical characteristics of the study participants according to sex. Smoking and alcohol consumption were significantly more common in men than in women (both *p* < 0.001). Furthermore, 34.5% (2.0) of men and 15.3% (1.2) of women were engaged in regular exercise; the difference was statistically significant (*p* < 0.001). However, there was no significant difference in the frequency of walking for physical activity between men and women (40.4% vs. 40.2%). Regarding body composition and bone mineral density measured by DEXA, the ASM index was higher in men than in women, but there was no difference in the prevalence of low skeletal muscle mass between the sexes. Osteopenia and osteoporosis of the lumbar spine were more common in women than in men (*p* < 0.001). Daily protein intake (g/kg/day) was lower in women than in men. Furthermore, low protein intake and LBP were significantly more common in women than in men (41.6% vs. 24.1% and 29.7% vs. 12.6%, respectively, both *p* < 0.001).

Table 2 shows the factors associated with LBP in men. Men with LBP were significantly older (*p* = 0.006) and more likely to smoke (*p* = 0.018) and have a lower body mass index (*p* < 0.001) and educational level (*p* = 0.002) than men without LBP. Men without LBP tended to engage in exercise more often than men with LBP (*p* = 0.001). Men without LBP also had a significantly higher ASM index (*p* = 0.003); however, there was no significant difference in the frequency of low skeletal muscle mass between those with and without LBP. Daily protein intake (g/kg/day) and the proportion with low protein intake were similar between the groups. OA in the lumbar spine was significantly more common in men with LBP than in those without LBP (*p* = 0.039).

Table 3 shows the factors associated with LBP in women. Women with LBP were significantly older (*p* < 0.001) and had a significantly lower educational level (*p* < 0.001) and lower household income (*p* = 0.024). Women with LBP were more likely to work in agriculture, forestry, fisheries, machine fitting, or simple labor (*p* = 0.004). Women without LBP tended to engage in exercise more often than women with LBP (*p* = 0.045). There was no significant difference in the mean ASM index value or the proportion with low skeletal muscle mass between those with and without LBP. Both daily protein intake (g/kg/day) and the proportion with low protein intake were significantly lower in women with LBP than in those without LBP (*p* = 0.011 and *p* = 0.008, respectively). Women with LBP had a higher prevalence of multiple comorbidities (*p* = 0.014) and OA in the lumbar spine than those without LBP (*p* < 0.001).

The results of multivariable logistic regression analysis of the association between protein intake, regular exercise, and LBP are presented in Table 4. In the subgroup analysis according to sex, the association between muscle strengthening exercise and LBP was significant in men (OR 2.34; 95% CI 1.24–4.44) but not in women (OR 0.89; 95% CI 0.51–1.55). Low protein intake (<0.8 g/kg/day) was associated with high odds for LBP only in women (OR 1.83; 95% CI 1.12–2.99).

Table 5 shows the association between combined protein intake, regular exercise, and LBP. After designating the participants having good protein intake in the exercise group as the reference group, it was found that the risk of LBP was higher in participants with a low protein intake and no exercise (OR 2.00; 95% CI 1.20–3.33) than in the reference group. The risk of LBP in the low protein intake with exercise group and good protein intake without exercise group was not significantly different from that in the reference group. When subgroup analysis was performed according to sex, women with low protein intake and no exercise had a higher risk of LBP (OR 2.91; 95% CI 1.48–5.72) than women in the reference group. However, the association was not significant in men (OR 1.55; 95% C, 0.72–3.34).

## 4. Discussion

This study evaluated the relationship between daily dietary protein intake, regular exercise, and LBP using nationally representative data from the KNHANES. Not performing muscle-strengthening exercise was associated with a higher risk of LBP in men, and low protein intake was associated with a higher risk of LBP in women.

Multiple factors can cause LBP, including structural changes in the lumbar spine and lifestyle, psychological, and social factors. Aerobic exercise plays a crucial role in relieving LBP by increasing blood flow and providing nutrients to the soft tissues in the lumbar structures, thereby improving the healing process in the damaged tissues and reducing stiffness [20]. In addition, physical activity, which can increase aerobic capacity and muscle strength, especially of the lumbar extensor muscles, assists patients with LBP in undertaking everyday activities [21]. Several studies have evaluated the effect of exercise in the aging population with LBP. Liu et al. and Jou et al. found that Tai Chi and core stabilization exercises reduced pain and protected neuromuscular function in the lower limbs in aging individuals (aged 50 years old or older) with LBP [22,23]. Our present findings are in line with the previous reports, i.e., regular exercise or walking is an important lifestyle factor that can prevent LBP in middle-aged and older adults.

In clinical practice, many older adults are vulnerable to LBP because of their inability to exercise enough to achieve the required clinical outcome. Age-related endocrine and metabolic alterations lead to changes in body composition, including progressive loss of muscle and bone mass and acquisition of fat mass [24]. Moreover, older adults have degenerative changes in the spine or multiple joints. The reduced walking speed is insufficient to rebuild muscles that undergo atrophy because of frailty, malnutrition, and anabolic resistance [25]. Protein intake has been shown to preserve muscle mass, prevent loss of physical function and prolong independent living in older adults [7,26]. However, in the real world, 7–41% of older adults are reported to have a daily protein intake lower than the RDA [27]. Over half of Korean adults over 60 years of age have a dietary protein intake that is lower than the RDA [28].

In our study, the probability of LBP was higher in women who had low protein intake and did not exercise than in their counterparts who had a good protein intake and exercised. Furthermore, 41.6% of women and 24.1% of men had a protein intake below the RDA (<0.8 g/kg/day). The marked association between protein intake and LBP in women may reflect their lower skeletal muscle mass relative to men. Although the impact of protein intake on LBP remains unclear, there are several plausible mechanisms. First, a prospective Women’s Health Initiative study that analyzed the data of 24,417 women found that a higher protein intake was associated with better preservation of muscle strength [29]. Low muscle strength is associated with an increased risk of LBP [30,31]. Second, protein intake may play a significant role in alleviating pain via muscle recovery. It was reported that the use of protein supplements reduces muscle damage and helps muscle recovery by remodeling skeletal muscle and is strongly recommended for muscle recovery after submaximal exercise [32]. Two recent randomized controlled trials examined the effects of amino acid or protein supplementation on joint pain [12,33]. Third, the protein source for muscle building was regarded as a promising factor. There was a sex difference in the association between each protein source and lean mass. The intake of total protein foods was positively associated with the appendicular lean mass index in both men and women, but consumption of seafood and plant protein foods were positively associated with appendicular lean mass index in women only [34]. Further studies are needed to investigate the impact of each protein source on LBP.

Although chronic pain is an important health issue in the older population, few studies have evaluated the effect of exercise or nutrition on LBP in aging populations. To the best of our knowledge, this is the first representative nationwide study to evaluate the combined associations between exercise, dietary protein intake, and LBP. We demonstrated that engagement in exercise and sufficient protein intake are associated with a low probability of LBP in middle-aged and older adults in the general Korean population.

This study had several limitations. First, the causal relationship between exercise and progression of symptoms was unclear because the KNHANES only records cross-sectional data. The American Physical Therapy Association recommends strengthening exercises and progressive walking in older patients with LBP [35] because regular exercise may help to relieve LBP and prevent further damage. Second, we did not have data on the history of pharmacological or surgical treatment of LBP in the study participants. To minimize the possible confounding effect of medical treatment on LBP, we excluded subjects with vertebral fracture and those with advanced arthritic changes in the lumbar spine as identified on plain radiography. Degeneration of the lumbar spine affects LBP, and an association between vertebral OA and LBP has already been reported [36,37]. These degenerative changes in the spine are highly likely to cause chronic intractable LBP that cannot be managed by lifestyle modifications, such as regular exercise and daily nutrient control; in such cases, timely medical intervention helps reduce the pain induced by severely progressive arthritis of the lumbar spine. Third, the KNHANES collected dietary data by 24 h recall. One day of data may not have accurately reflected the average amount of nutrients ingested by the study participants. However, to increase the accuracy of this large population-based survey, the frequency of food intake and portion size for each item were estimated using the semi-quantitative food intake frequency survey table, which verified the validity of 112 food items, and the food intake frequency survey, which consists of 63 food items. Furthermore, trained dietitians helped the study participants recall their dietary information. A further prospective study over a longer period will be needed to evaluate the association between regular exercise, dietary protein intake, and LBP in the older population.

## 5. Conclusions

In this study, we found that a combination of sufficient dietary protein intake (≥0.8 g/kg/day) and regular exercise was associated with a low probability of LBP in middle-aged and older Korean adults. Regular exercise and a daily dietary protein intake equivalent to the RDA should be maintained in everyday life to prevent LBP. Women who are middle-aged or older who cannot exercise regularly, including walking as a physical activity, should be encouraged to consume adequate dietary protein to reduce their risk of LBP.

## Figures and Tables

**Table 1:** Characteristics of study participants by sex.

Variable	Men (n = 1047)	Women (n = 1320)	*p*-Value
Age (years)	59.4 (0.34)	59.7 (0.30)	0.620
Body mass index (kg/m^2^)	23.9 (0.12)	24.3 (0.11)	0.031
Educational level			<0.001
≤Elementary school	28.9% (2.1)	53.3% (2.1)	
Middle and high school	52.2% (2.1)	40.3% (1.9)	
≥College	18.9% (1.8)	6.3% (0.9)	
Occupation			<0.001
Office work	12.4% (1.2)	3.4% (0.5)	
Sales and services	11.0% (1.3)	13.9% (1.2)	
Agriculture, forestry, and fisheries	20.7% (2.5)	11.9% (1.7)	
Machine fitting and simple labor	28.2% (1.9)	15.9% (1.2)	
Unemployed	27.7% (1.5)	55.0% (1.9)	
Household income			0.192
Low	26.6% (1.9)	24.1% (1.6)	
Lower middle	26.7% (1.7)	24.4% (1.7)	
Upper middle	24.9% (1.6)	28.5% (1.5)	
High	21.8% (1.6)	23.0% (1.7)	
Smoking status			<0.001
Ex-smoker	50.4% (2.0)	2.8% (0.7)	
Current smoker	35.7% (1.8)	3.6% (0.7)	
Alcohol consumption	71.5% (1.8)	30.5% (1.6)	<0.001
Muscle strengthening exercise ^a^	34.5% (2.0)	15.3% (1.2)	<0.001
Walking for physical activity ^b^	40.4% (1.8)	40.2% (1.7)	0.935
DEXA			
Trunk lean mass (kg)	24.69 (0.15)	18.50 (0.09)	<0.001
Appendicular skeletal muscle mass/height^2^	7.48 (0.04)	5.86 (0.03)	<0.001
Low skeletal muscle mass ^c^	28.8% (2.1)	25.4% (1.8)	0.146
Lumbar spine BMD			<0.001
Normal	59.0% (1.9%)	28.1% (1.7%)	
Osteopenia	35.6% (1.8%)	45.9% (2.0%)	
Osteoporosis	5.4% (0.8%)	26.0% (1.8%)	
Protein intake (g/day)	81.0 (1.41)	55.8 (1.12)	<0.001
Protein intake (g/kg/day)	1.22 (0.02)	0.98 (0.02)	<0.001
Low protein intake < 0.8 g/kg/day	24.1% (1.6)	41.6% (1.7)	<0.001
Total energy intake (kcal/day)	2320.1 (34.54)	1643.5 (28.74)	<0.001
Comorbidity ^d^			0.176
None	45.8% (2.3)	41.4% (1.9)	
1–2	36.6% (2.0)	39.0% (1.5)	
≥3	17.6% (1.6)	19.7% (1.5)	
Lumbar spine osteoarthritis			0.120
Normal	24.3% (1.9)	28.0% (1.8)	
Grade 1	75.7% (1.9)	72.0% (1.8)	
Low back pain	12.6% (1.6)	29.7% (1.9)	<0.001

Values are presented as the mean ± standard error or estimated percentage (standard error). ^a^ Muscle strengthening exercises (push-ups, sit-ups, and lifting dumbbells or weights) for at least two days a week; ^b^ walking for at least 30 min for five days a week; ^c^ low skeletal muscle mass (the 2019 Asian Working Group for Sarcopenia consensus statement defines the cut-off values for a diagnosis of low muscle mass as sarcopenia is <7.0 kg/m^2^ in men and <5.4 kg/m^2^ in women by DEXA); ^d^ comorbidity (hypertension, diabetes mellitus, chronic kidney disease, rheumatoid arthritis, cancer, liver cirrhosis, and depression). BMD, bone mineral density; DEXA, dual-energy X-ray absorptiometry.

**Table 2:** Factors associated with low back pain in men.

Variable	Low Back Pain		*p*-Value
	Yes (n = 127)	No (n = 920)	
Age (years)	61.6 (0.90)	59.1 (0.33)	0.006
Body mass index (kg/m^2^)	23.0 (0.23)	24.0 (0.13)	<0.001
Educational level			0.002
≤Elementary school	45.3% (6.0)	26.5% (2.1)	
Middle and high school	35.8% (5.7)	54.6% (2.0)	
≥College	18.9% (4.6)	18.9% (1.9)	
Occupation			0.202
Office work	13.7% (4.0)	15.2% (1.6)	
Sales and services	6.9% (3.5)	13.3% (1.9)	
Agriculture, forestry, and fisheries	24.9% (6.1)	16.5% (2.5)	
Machine fitting and simple labor	24.7% (5.3)	31.4% (2.5)	
Unemployed	29.8% (4.9)	23.6% (1.8)	
Household income			0.313
Low	30.5% (5.2)	26.0% (2.0)	
Lower middle	29.9% (5.6)	26.3% (1.8)	
Upper middle	16.8% (4.0)	26.0% (1.8)	
High	22.8% (3.7)	21.7% (1.7)	
Smoking status			0.018
Ex-smoker	57.9% (5.7)	49.3% (2.2)	
Current smoker	38.2% (5.7)	35.4% (2.0)	
Alcohol consumption	72.9% (4.5)	71.3% (2.0)	0.742
Muscle strengthening exercise ^a^	19.3% (3.9)	36.7% (2.2)	0.001
Walking for physical activity ^b^	42.0% (5.1)	40.2% (2.0)	0.743
DEXA			
Trunk lean mass (kg)	23.97 (0.32)	24.79 (0.16)	0.015
Appendicular skeletal muscle mass/height^2^	7.26 (0.08)	7.51 (0.04)	0.003
Low skeletal muscle mass ^c^	30.6% (5.1)	28.5% (2.1)	0.695
Lumbar spine BMD			0.078
Normal	48.4% (5.5)	60.5% (1.9)	
Osteopenia	44.8% (5.5)	34.3% (1.8)	
Osteoporosis	6.8% (2.5)	5.2% (0.8)	
Protein intake (g/day)	75.2 (3.55)	81.8 (1.50)	0.083
Protein intake (g/kg/day)	1.17 (0.06)	1.23 (0.02)	0.382
Low protein intake < 0.8 g/kg/day	27.9% (3.6)	23.6% (1.7)	0.267
Total energy intake (kcal/day)	2126.5 (65.47)	2348.0 (37.55)	0.003
Comorbidity ^d^			0.717
None	48.8% (6.1)	45.3% (2.4)	
1–2	32.8% (4.4)	37.2% (2.2)	
≥3	18.4% (4.2)	17.5% (1.6)	
Lumbar spine osteoarthritis			0.039
Normal	15.2% (3.9)	25.6% (2.1)	
Grade 1	84.8% (3.9)	74.4% (2.1)	

Values are presented as the mean ± standard error or as the estimated percentage (standard error). ^a^ Muscle strengthening exercises (push-ups, sit-ups, and lifting dumbbells or weights) for at least two days a week; ^b^ walking for at least 30 min for five days a week; ^c^ low skeletal muscle mass (the 2019 Asian Working Group for Sarcopenia consensus statement states the cut-off value for a diagnosis of low muscle mass as sarcopenia is <7.0 kg/m^2^ in men by DEXA); ^d^ comorbidity (hypertension, diabetes mellitus, chronic kidney disease, rheumatoid arthritis, cancer, liver cirrhosis, and depression). BMD, body mass index; DEXA, dual-energy X-ray absorptiometry.

**Table 3:** Factors associated with low back pain in women.

Variable	Low Back Pain		*p*-Value
	Yes (n = 380)	No (n = 940)	
Age (years)	61.6 (0.63)	58.8 (0.32)	<0.001
BMI (kg/m^2^)	24.5 (0.23)	24.2 (0.12)	0.222
Educational level			<0.001
≤Elementary school	67.2% (3.3)	47.5% (2.6)	
Middle and high school	29.3% (3.0)	45.0% (2.4)	
≥College	3.5% (1.1)	7.5% (1.2)	
Occupation			0.004
Office work	1.5% (0.6)	5.7% (0.9)	
Sales and services	17.3% (2.4)	17.5% (1.8)	
Agriculture, forestry, and fisheries	15.4% (2.8)	9.3% (2.5)	
Machine fitting and simple labor	19.3% (2.9)	15.8% (1.6)	
Unemployed	46.4% (3.5)	51.7% (2.5)	
Household income			0.024
Low	29.9% (2.9)	21.6% (1.8)	
Lower middle	23.7% (2.9)	24.7% (2.2)	
Upper middle	29.1% (2.8)	28.3% (1.9)	
High	17.2% (2.4)	25.4% (2.2)	
Smoking status			0.802
Ex-smoker	2.5% (0.9)	2.9% (0.8)	
Current smoker	4.1% (1.4)	3.4% (0.7)	
Alcohol consumption	26.9% (3.0)	32.1% (1.9)	0.149
Muscle strengthening exercise ^a^	11.4% (1.9)	16.9% (1.6)	0.045
Walking for physical activity ^b^	36.6% (3.1)	41.7% (2.2)	0.212
DEXA			
Trunk lean mass (kg)	18.54 (0.16)	18.49 (0.10)	0.753
Appendicular skeletal muscle mass/height^2^	5.92 (0.05)	6.32 (0.09)	0.107
Low skeletal muscle mass ^c^	22.2% (2.4)	26.7% (2.1)	0.123
Lumbar spine BMD			0.097
Normal	24.9% (2.9)	29.5% (2.0)	
Osteopenia	44.0% (3.5)	46.7% (2.3)	
Osteoporosis	31.1% (2.0)	23.8% (2.2)	
Protein intake (g/day)	51.9 (1.76)	53.9 (2.38)	0.015
Protein intake (g/kg/day)	0.91 (0.03)	0.93 (0.04)	0.011
Low protein intake < 0.8 g/kg/day	49.1% (3.5)	38.5% (1.8)	0.008
Total energy intake (kcal/day)	1605.6 (46.94)	1659.6 (30.82)	0.269
Comorbidity ^d^			0.014
None	34.4% (2.4)	44.3% (2.3)	
1–2	40.6% (2.9)	38.3% (2.0)	
≥3	25.0% (2.9)	17.4% (1.6)	
Lumbar spine osteoarthritis			<0.001
Normal	17.6% (2.6)	32.4% (2.3)	
Grade 1	82.4% (2.6)	67.6% (2.3)	

Values are presented as the mean ± standard error or as the estimated percentage (standard error). ^a^ Muscle strengthening exercises (push-ups, sit-ups, and lifting dumbbells or weights) for at least two days a week; ^b^ walking for at least 30 min for five days a week; ^c^ low skeletal muscle mass (the 2019 Asian Working Group for Sarcopenia consensus statement states the cut-off value for a diagnosis of low muscle mass as sarcopenia is <5.4 kg/m^2^ in women by DEXA); ^d^ comorbidity (hypertension, diabetes mellitus, chronic kidney disease, rheumatoid arthritis, cancer, liver cirrhosis, and depression). BMD, body mass index; DEXA, dual-energy X-ray absorptiometry.

**Table 4:** Association between protein intake, regular exercise, and low back pain.

	Crude		Model 1 ^a^		Model 2 ^b^		Model 3	
	OR	95% CI	OR	95% CI	OR	95% CI	OR	95% CI
Total study population								
Protein intake < 0.8 g/kg/day	1.88	(1.42–2.49)	1.46	(1.09–1.95)	1.44	(1.05–1.96)	1.32 ^c^	(0.90–1.92)
Protein intake ≥ 0.8 g/kg/day	1		1		1		1	
Muscle strengthening exercise (-)	2.38	(1.60–3.53)	1.69	(1.15–2.48)	1.48	(1.01–2.17)	1.43 ^d^	(0.95–2.16)
Muscle strengthening exercise (+)	1		1		1		1	
Walking for physical activity (-)	1.13	(0.84–1.51)	1.16	(0.85–1.58)	1.16	(0.84–1.60)	1.18 ^e^	(0.84–1.66)
Walking for physical activity (+)	1		1		1		1	
Men								
Protein intake < 0.8 g/kg/day	1.33	(0.87–2.01)	1.24	(0.80–1.90)	1.32	(0.82–2.11)	1.07 ^c^	(0.59–1.96)
Protein intake ≥ 0.8 g/kg/day	1		1		1		1	
Muscle strengthening exercise (-)	2.57	(1.43–4.59)	2.45	(1.35–4.55)	2.29	(1.23–4.24)	2.34 ^d^	(1.24–4.44)
Muscle strengthening exercise (+)	1		1		1		1	
Walking for physical activity (-)	0.92	(0.55–1.53)	0.95	(0.57–1.61)	0.93	(0.54–1.60)	0.76 ^e^	(0.42–1.39)
Walking for physical activity (+)	1		1		1		1	
Women								
Protein intake < 0.8 g/kg/day	1.68	(1.16–2.43)	1.59	(1.08–2.32)	1.57	(1.04–2.36)	1.83 ^c^	(1.12–2.99)
Protein intake ≥ 0.8 g/kg/day	1		1		1		1	
Muscle strengthening exercise (-)	1.24	(0.74–2.10)	1.16	(0.70–1.93)	0.95	(0.58–1.54)	0.89 ^d^	(0.51–1.55)
Muscle strengthening exercise (+)	1		1		1		1	
Walking for physical activity (-)	1.26	(0.87–1.85)	1.29	(0.89–1.88)	1.32	(0.89–1.95)	1.53 ^e^	(0.99–2.35)
Walking for physical activity (+)	1		1		1		1	

^a^ Model 1: age, sex; ^b^ Model 2: age, sex, BMI, smoking, alcohol consumption, education, occupation, household income; ^c^ Model 3: age, sex, BMI, smoking, alcohol consumption, education, occupation, household income, osteoporosis of the lumbar spine, severity of lumbar osteoarthritis, comorbidity, total energy intake, muscle strengthening exercise, walking for physical activity; ^d^ Model 3: age, sex, BMI, smoking, alcohol consumption, education, occupation, household income, osteoporosis of the lumbar spine, severity of lumbar osteoarthritis, comorbidity, protein intake, total energy intake, walking for physical activity; ^e^ Model 3: age, sex, BMI, smoking, alcohol consumption, education, occupation, household income, osteoporosis of the lumbar spine, severity of lumbar osteoarthritis, comorbidity, protein intake, total energy intake, muscle strengthening exercise. BMI, body mass index; CI, confidence interval; OR, odds ratio.

**Table 5:** Association between combined protein intake, regular exercise, and low back pain.

	Crude		Model 1 ^b^		Model 2 ^c^		Model 3 ^d^	
	OR	95% CI	OR	95% CI	OR	95% CI	OR	95% CI
Total study population								
Low protein intake and exercise ^a^ (-)	2.62	(1.84–3.74)	1.86	(1.29–2.67)	1.99	(1.27–3.11)	2.00	(1.20–3.33)
Low protein intake and exercise (+)	1.35	(0.93–1.96)	1.01	(0.69–1.50)	1.11	(0.72–1.72)	0.97	(0.58–1.63)
Good protein intake and exercise (-)	1.38	(1.004–1.91)	1.26	(0.92–1.74)	1.19	(0.82–1.73)	1.17	(0.78–1.75)
Good protein intake and exercise (+)	1		1		1		1	
Men								
Low protein intake and exercise (-)	2.08	(1.23–3.50)	1.83	(1.06–3.15)	1.93	(1.04–3.59)	1.55	(0.72–3.34)
Low protein intake and exercise (+)	0.92	(0.43–1.96)	0.83	(0.38–1.82)	1.06	(0.44–2.54)	0.89	(0.35–2.26)
Good protein intake and exercise (-)	1.32	(0.77–2.26)	1.37	(0.80–2.34)	1.27	(0.66–2.46)	1.18	(0.63–2.24)
Good protein intake and exercise (+)	1		1		1		1	
Women								
Low protein intake and exercise (-)	2.08	(1.32–3.28)	1.87	(1.17–2.96)	2.06	(1.15–3.67)	2.91	(1.48–5.72)
Low protein intake and exercise (+)	1.23	(0.80–1.88)	1.09	(0.69–1.70)	1.24	(0.74–2.08)	1.36	(0.72–2.54)
Good protein intake and exercise (-)	1.24	(0.84–1.84)	1.21	(0.82–1.78)	1.16	(0.73–1.86)	1.21	(0.70–2.08)
Good protein intake and exercise (+)	1		1		1		1	

^a^ Low protein intake, <0.8 g/kg/day; good protein intake, ≥0.8 g/kg/day; exercise, muscle-strengthening exercise or walking for physical activity. ^b^ Model 1: age, sex; ^c^ Model 2: age, sex, BMI, smoking, alcohol consumption, education, occupation, household income; ^d^ Model 3: age, sex, BMI, smoking, alcohol consumption, education, occupation, household income, lumbar spine osteoporosis, the severity of lumbar OA, comorbidity, total energy intake. BMI, body mass index; OR, odds ratio; 95% CI, 95% confidence interval; OA, osteoarthritis.

## Data Availability

The KNHANES data can be downloaded from the website (https://knhanes.kdca.go.kr/knhanes/main.do (accessed on 21 December 2021).
